# The effect of curcumin supplementation on cognitive function: an updated systematic review and meta-analysis

**DOI:** 10.3389/fnut.2025.1549509

**Published:** 2025-04-16

**Authors:** Wenlong Wang, Rui Zhao, Bingzheng Liu, Kelei Li

**Affiliations:** ^1^Institute of Brain Science and Brain-Inspired Research, Shandong First Medical University and Shandong Academy of Medical Sciences, Jinan, Shandong, China; ^2^Institute of Nutrition and Health, Qingdao University, Qingdao, China

**Keywords:** curcumin, cognition, Alzheimer’s disease, meta-analysis, RCT

## Abstract

**Background:**

Previous randomized controlled trials (RCTs) did not draw a consistent conclusion about the effect of curcumin on cognitive function.

**Methods:**

We searched Web of Science, PubMed, Cochrane Library and Embase, and 9 RCTs (including 12 independent comparisons) with 501 subjects were included in the present meta-analysis.

**Results:**

Compared with placebo, supplementation of curcumin significantly improved global cognitive function (SMD, 0.82; 95% CI, 0.19 to 1.45; *p* = 0.010). A curvilinear dose–response effect was observed, and the optimal dose is 0.8 g/day. Subgroup analysis indicated that the beneficial effect of curcumin on cognition was significant only if duration ≥24 weeks (SMD, 1.15; 95% CI, 0.13 to 2.18; *p* = 0.027), age of participants ≥60 years (SMD, 1.12; 95% CI, 0.03 to 2.21; *p* = 0.044), or participants from Asian countries (SMD, 0.96; 95% CI, 0.08 to 1.83; *p* = 0.032). Otherwise, this effect became non-significant (*p* > 0.05). Sensitivity analysis by excluding each study one by one or excluding all studies with high risk of bias did not obviously influence the final results. No significant publication bias was observed (P for Begg’s Test and Egger’s test = 0.150 and 0.493, respectively).

**Conclusion:**

Supplementation of curcumin can effectively improve global cognitive function, and the optimal dose and duration is 0.8 g/day and ≥24 weeks. The beneficial effect of curcumin on cognition is more potent in older and Asian participants than younger and Western ones.

## Introduction

Alzheimer’s disease (AD) is rapidly becoming one of the most expensive, burdening, and deadly diseases of this century (1). Oxidative stress and neuroinflammation are involved in cognitive impairment and AD pathology (2, 3). In recent years, the beneficial effect of polyphenols on cognition has been paid much attention due to its good antioxidant and anti-inflammatory activities (4–6).

Curcumin is an orange-yellow colored, lipophilic polyphenol substance in Turmeric (*Curcuma longa*) (7). Cohort studies indicated that curcumin intake was associated with improved global cognitive function (8, 9). Several randomized controlled trials (RCTs) evaluated the effect of curcumin on cognition, but the results were inconsistent (10–17). Two previous meta-analyses of RCTs explored the effect of curcumin on cognition in 2019 and 2021 (18, 19). Their overall effect sizes about global cognition were non-significant, and significant beneficial effect of curcumin was only observed for cognitive subdomain “working memory” and subpopulation “older subjects” (18, 19). Since 2021, several new RCTs have been published (20–23). Therefore, we conducted an updated meta-analysis to evaluate the effect of curcumin on global cognition and explore the dose–response relationship.

## Materials and methods

### Data sources and study selection

We searched Web of Science, PubMed, Cochrane Library and Embase for terms cognitive OR cognition OR memory OR Alzheimer’s disease OR dementia, combined with curcumin OR curcuminoid OR curcuma OR turmeric OR diferuloylmethane, up to September 2024. For inclusion, studies had to fulfill the following criteria: had a randomized placebo controlled (RCT) design; evaluated the effect of curcumin supplementation on cognitive function; reported data of global cognitive function. Studies were excluded if allocation of participants to the treatments was not randomized; data indispensable for a meta-analysis were not reported; the effect of curcumin could not be separated from other active ingredients; studies did not have a placebo control group. Hand searching of reference of all relevant articles and reviews was undertaken. The review was not registered before and no protocol was prepared.

### Data extraction and quality assessment

Study selection and data extraction were undertaken independently by two investigators, with discrepancies resolved by consensus. The data collected included the first author’s name, year of publication, sample size, mean age, sex ratio (male/total subjects) and healthy status of subjects, study design, daily dose and product information of curcumin, duration of intervention, mean and corresponding standard deviation (SD) of global cognition score, method for evaluating global cognitive function.

Cochrane criteria was used to assess the quality of included studies, including sequence generation, allocation concealment, blinding of participants, personnel and outcome assessors, incomplete outcome data and selective outcome reporting (24).

### Statistical methods

Changes from baseline to endpoint and corresponding SD were used for data analysis. If SD of change was not reported, they were imputed based on SD at baseline and endpoint according to the Cochrane Handbook for Systematic Reviews of Interventions (24). For studies with two or more intervention groups sharing one control group, we separated the shared control group into two or more groups (the number was the same as intervention groups) and included these comparisons into meta-analysis as if they were from different studies (24, 25).

All data analyses were conducted in Stata/SE 11.0 software (StataCorp, College Station, TX). Standard mean difference (SMD) was used as the effect size. A random-effect model was used to pool study-specific effect sizes which were visualized in a forest plot, and a *p*-value <0.05 was considered to be statistically significant. Heterogeneity was assessed by the chi-square method, and *I*^2^ > 50% indicated significant heterogeneity. Meta-regression was used to explore whether there is a linear relationship between daily dose of curcumin and effect size. Restricted cubic spline analysis (3 knots) was used to analyze curvilinear relationship between daily dose of curcumin and effect size. Subgroup analysis was undertaken to explore the sources of heterogeneity and their influence on effect size according to the difference in duration (≥24 weeks or <24 weeks), age (≥60 years or <60 years), percentage of male (≥50% or <50%), healthy status (AD or not), country (Asian or Western countries; Western countries include the United States and Australia, and no included study was conducted in Europe), methods for evaluating cognitive function. Funnel plot, Begg’s test and Egger’s test were used to assess publication bias. If significant publication bias was observed, trim-and-fill method was used to adjust the pooled effect size. Sensitivity analysis was conducted as follows: if one study evaluated global cognition by two or more different methods, data from one method was used to pool effect size, and then it was replaced with data generated by another method to see the influence on the final result; excluding each study one by one and reanalyzed the data; excluding studies having a high risk of bias at one or more validity criteria and then reanalyzing the remaining data. A *p* < 0.05 was considered to be statistically significant.

## Results

### Characteristics of included studies

**Figure 1:**
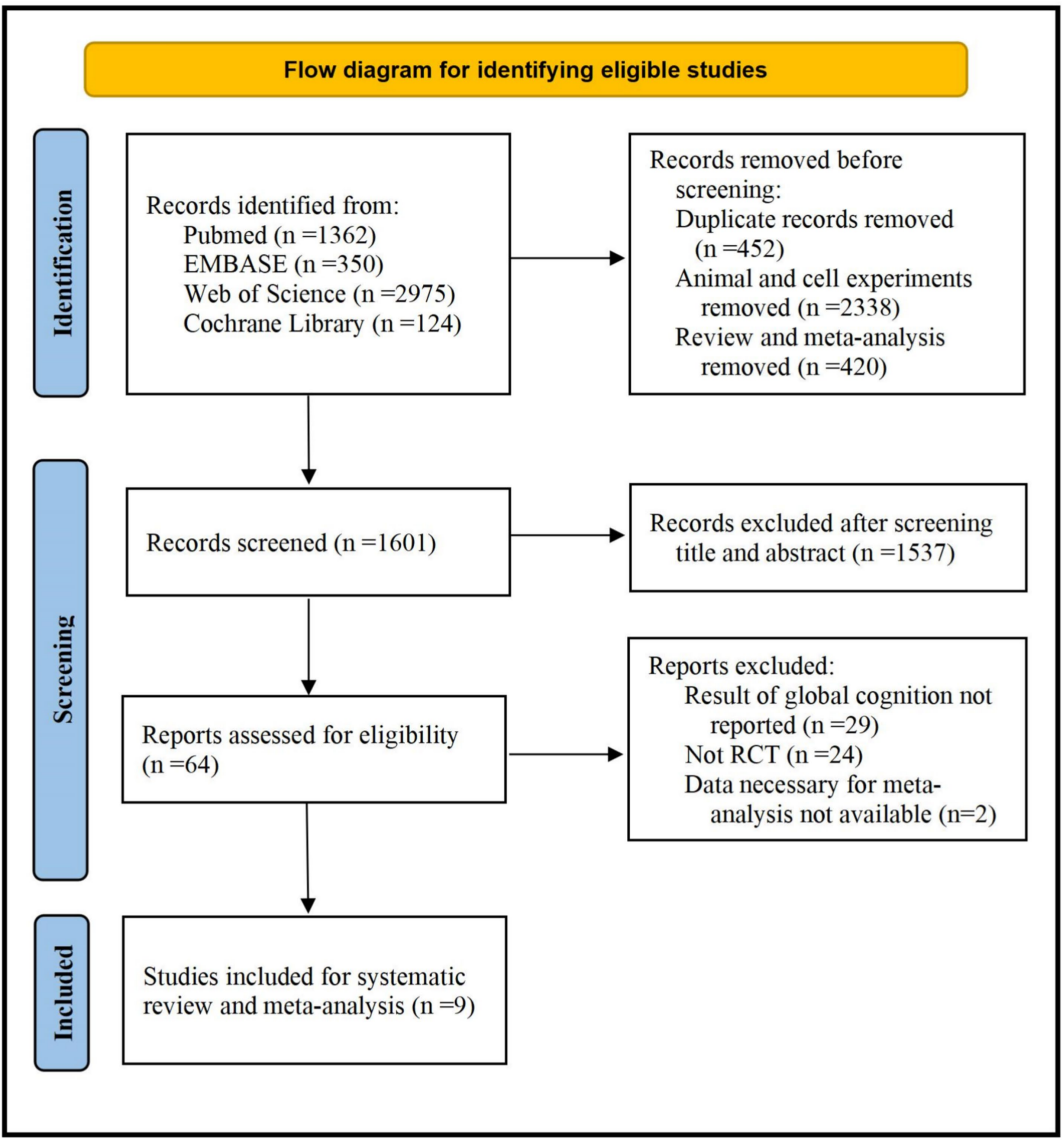
Flow diagram for identifying eligible studies.

A total of 4,811 studies were identified by electronic searches. Among these studies, 9 RCTs including 12 independent comparisons in 501 subjects were included in the present studies (10, 13–16, 21–23, 26). Detailed process of study selection was shown in Figure 1.

Characteristics of included studies of included studies were shown in Table 1. Three studies including six independent comparisons recruited AD patients, (10, 16, 22) two studies were conducted in older adults (14, 15), two studies included subjects with Schizophrenia (13, 26), and the remaining two studies were conducted in women with premenstrual syndrome and dysmenorrhea or subjects with chemotherapy-induced cognitive impairment (21, 23). Methods for evaluating global cognitive function including MMSE, MCCB, ADAS-Cog, MoCA, CAQ and NIH toolbox. The daily dose ranged from 0.16 to 4 g/day. The duration of five studies including 8 independent comparisons was more than 24 weeks (10, 15, 16, 22, 23). Four studies including six independent comparisons were conducted in Asian countries, and the remaining studies were conducted in Western countries. The average age of subjects ranged from 20.8 to 73.85 years. The percentage of male ranged from 0 to 75%.

**Table 1:** Characteristics of included studies.

Study	Healthy status of subjects	Sample size		Global cognition assessment	Intervention		Design	Dose (g/day)	Duration (week)	Country	Age (Year)	Male (%)
		T	C		T	C						
Baum et al. (2008) (HD) (10)	Alzheimer’s disease	11	4	MMSE	Curcumin capsule or powder	Placebo	RCT, double-blind	4	24	China	73.4	25.9
Baum et al. (2008) (LD) (10)	Alzheimer’s disease	8	4	MMSE	Curcumin capsule or powder	Placebo	RCT, double-blind	1	24	China	73.4	25.9
Kucukgoncu et al. (2019) (13)	Schizophrenia	5	5	MCCB	Theracurmin®*	Placebo	RCT, double-blind	0.18	8	United States	41.33	75
Ringman et al. (2012) (HD) (16)	Alzheimer’s disease	10	6	ADAS-Cog	Curcumin C3 Complex®	Placebo	RCT, double-blind	4	24	United States	73.85	36.7
Ringman et al. (2012) (LD) (16)	Alzheimer’s disease	9	5	ADAS-Cog	Curcumin C3 Complex®	Placebo	RCT, double-blind	2	24	United States	73.85	36.7
Rainey-Smith et al. (2016) (15)	Healthy older adults	39	57	MoCA	BCM-95®CG*	Placebo	RCT, double-blind	1.5	48	Australia	66	29.2
Wynn et al. (2018) (26)	Schizophrenia	17	19	MCCB	Theracumin®*	Placebo	RCT, double-blind	0.36	8	United States	50.5	83.3
Bahrami et al. (2023) (21)	Women with premenstrual syndrome and dysmenorrhea	57	60	CAQ	Curcuminoids and piperine*	Placebo	RCT, double-blind	0.5	12	Iran	20.8	0
Das et al. (2023) (CGM) (22)	Alzheimer’s disease	16	7	MMSE	Curcumin-galactomannan complex (CGM)*	Placebo	RCT, double-blind	0.8	24	India	64.58	68.8
Das et al. (2023) (USC) (22)	Alzheimer’s disease	15	6	MMSE	Unformulated standard curcumin complex (USC)	Placebo	RCT, double-blind	0.8	24	India	64.58	68.8
Putri Laksmidewi et al. (2024) (23)	Chemotherapy- induced cognitive impairment	39	39	MoCA	Curcumin caplets	Placebo	RCT, double-blind	0.4	32	Indonesia	48.6	NA
Kuszewski et al. (2020) (14)	Overweight or Obesity	31	32	NIH toolbox	Longvida®	Placebo	RCT, double-blind	0.16	16	Australia	65.75	46

*, curcumin product with enhanced bioavailability during preparation.HD, high dose; LD, low dose; T, treatment; C, control; RCT, randomized controlled trial; MMSE, Mini-Mental State Exam; MCCB, MATRICS Consensus Cognitive Battery; ADAS-Cog, Alzheimer’s Disease Assessment Scale-Cognitive Subscale; MoCA, Montreal Cognitive Assessment; CAQ, cognitive abilities questionnaire; NIH, National Institute of Health; NA, not available.

### Quality assessment

**Figure 2:**
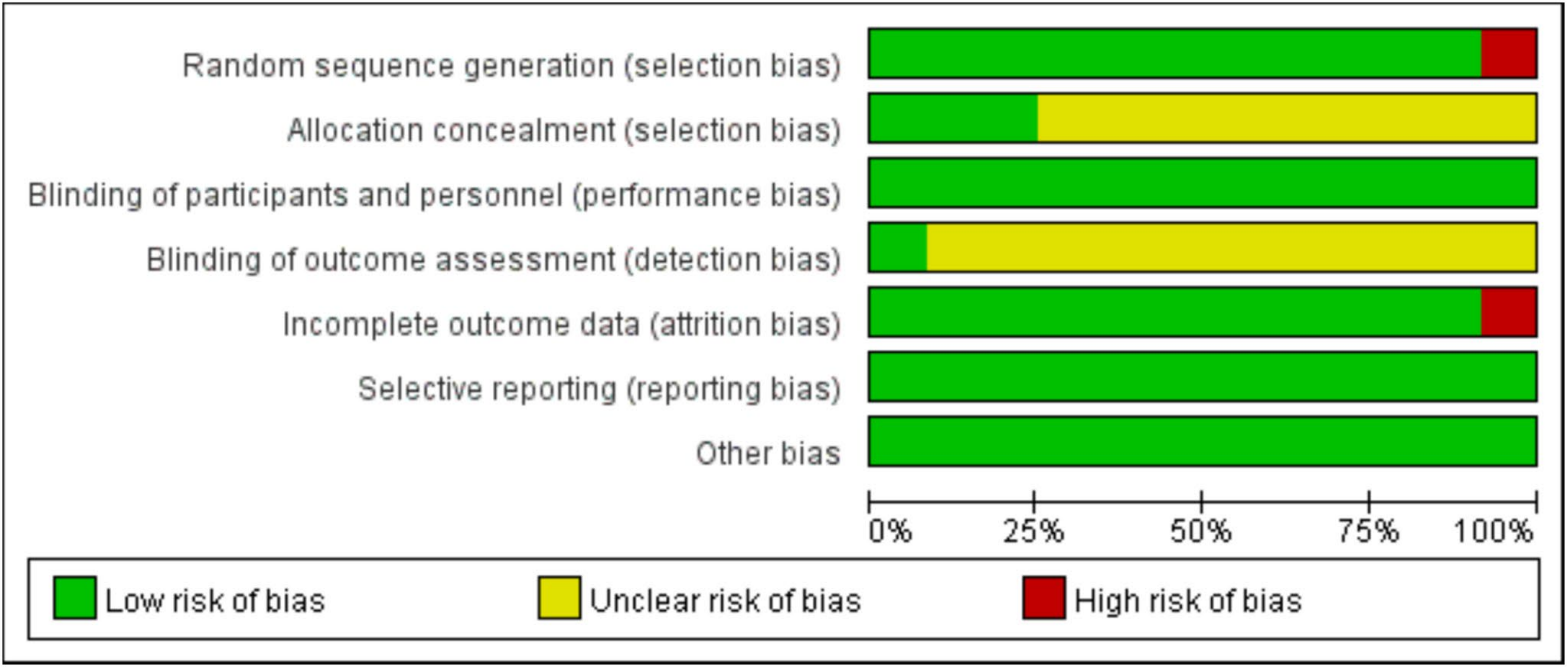
Judgments about each risk of bias item presented as percentages across all included studies.

**Figure 3:**
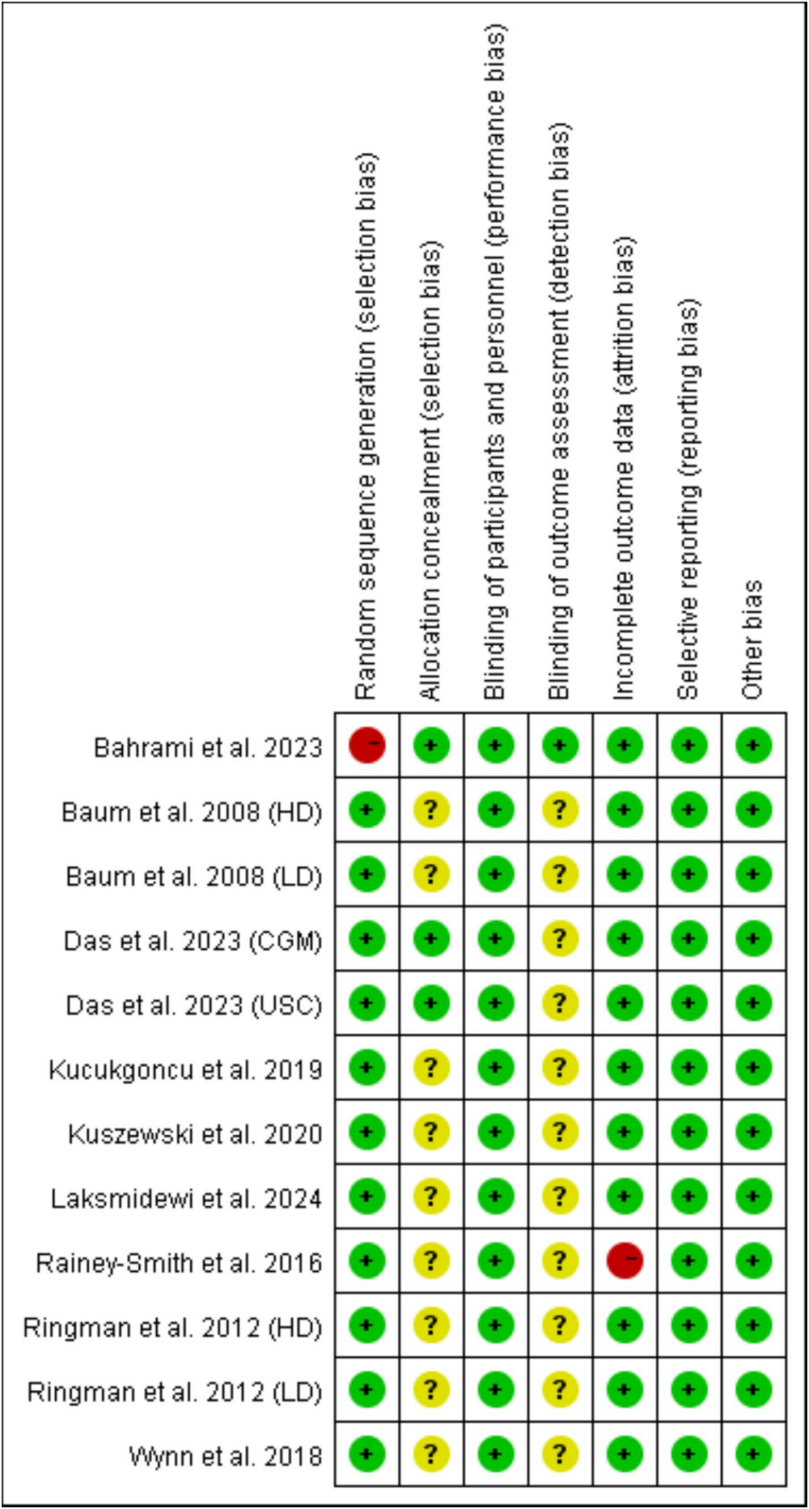
Judgments about each risk of bias item for each included study. Red ball, high risk of bias; yellow ball, unclear risk of bias; green ball, no risk of bias.

Random sequence generation (selection bias) and incomplete outcome data (attrition bias) were the major sources of bias, which was found in the study by Bahrami et al. (21) and the study by Rainey-Smith et al. (15), respectively (Figures 2, 3). For most of included studies, it was unclear whether randomized sequence could be foreseen by participants and investigators (allocation concealment) and whether assessors were blinded to intervention assignment (detection bias).

### The overall effect of curcumin on global cognitive function

**Figure 4:**
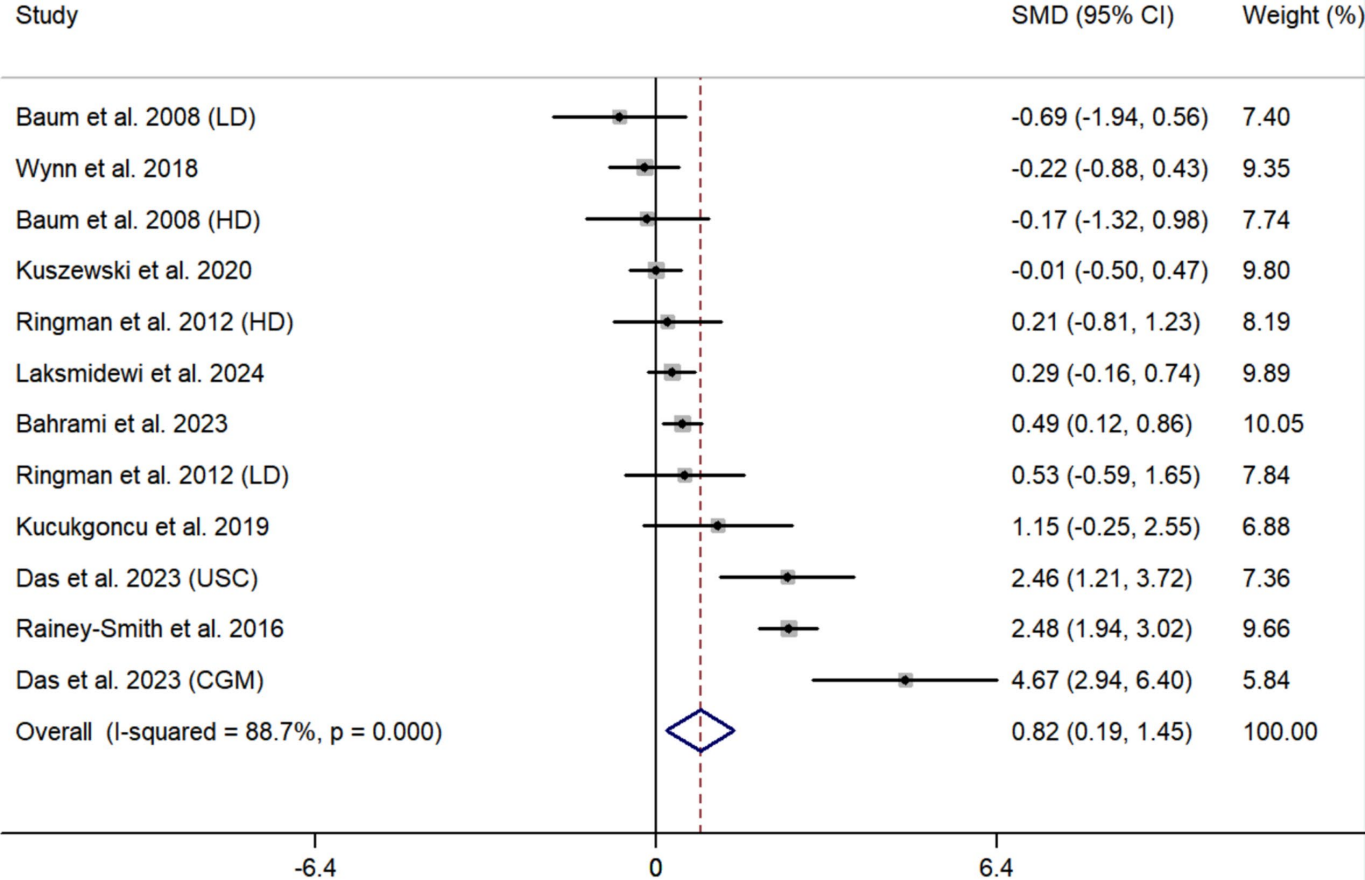
Pooled effect size of curcumin on global cognition.

Supplementation of curcumin significantly improved global cognitive function, and pooled effect size was 0.82 (95% CI, 0.19–1.45; *p* = 0.010) (Figure 4). However, significant heterogeneity was observed (*I*^2^ = 88.7%, *p* < 0.001).

### Dose–response analysis

**Figure 5:**
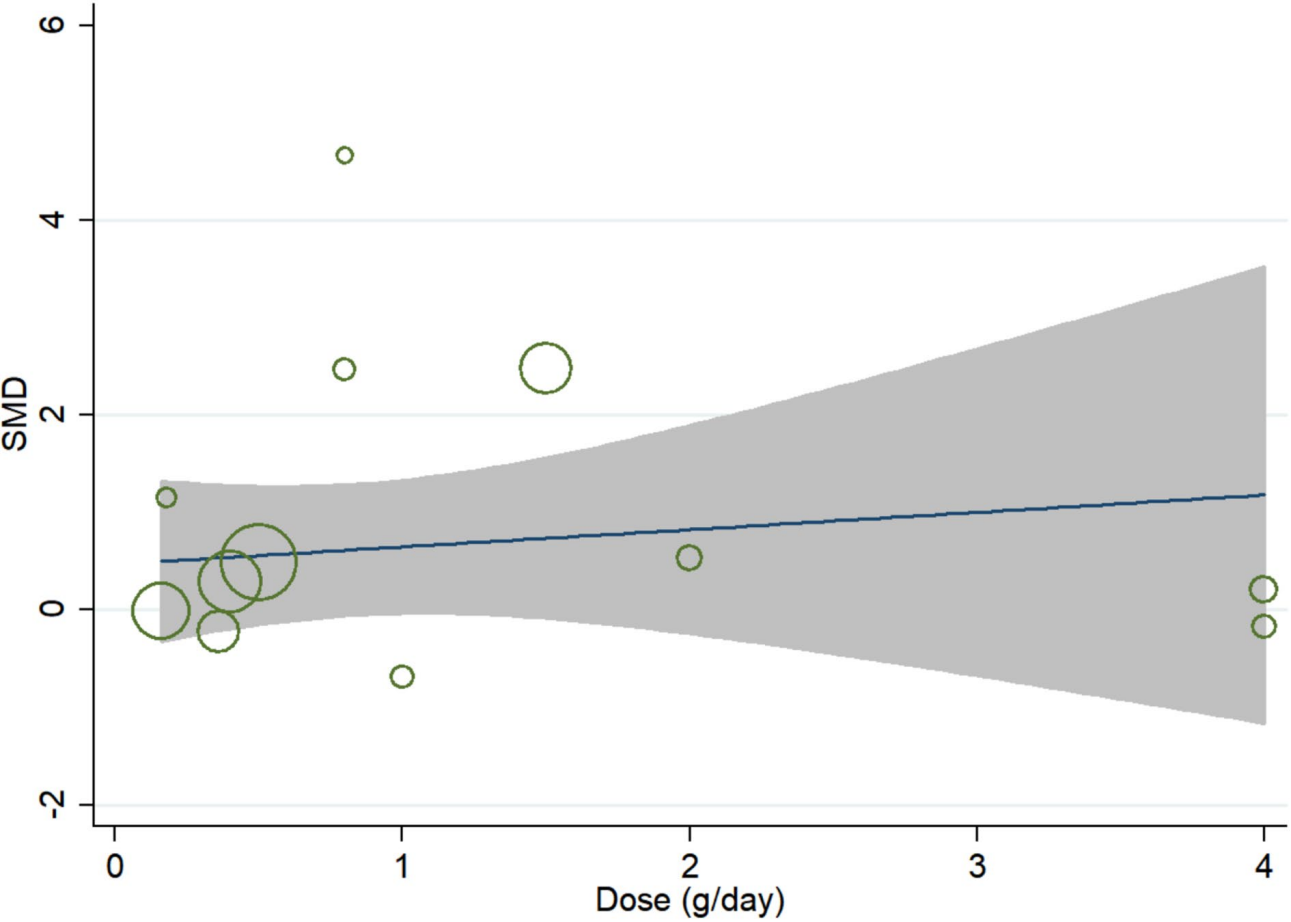
Meta-regression for linear dose–response analysis.

In meta-regression analysis, no significant linear relationship was observed between daily dose of curcumin and effect size. The coefficient was −0.16 (95% CI, −0.91 to 0.58; *p* = 0.636) (Figure 5).

**Figure 6:**
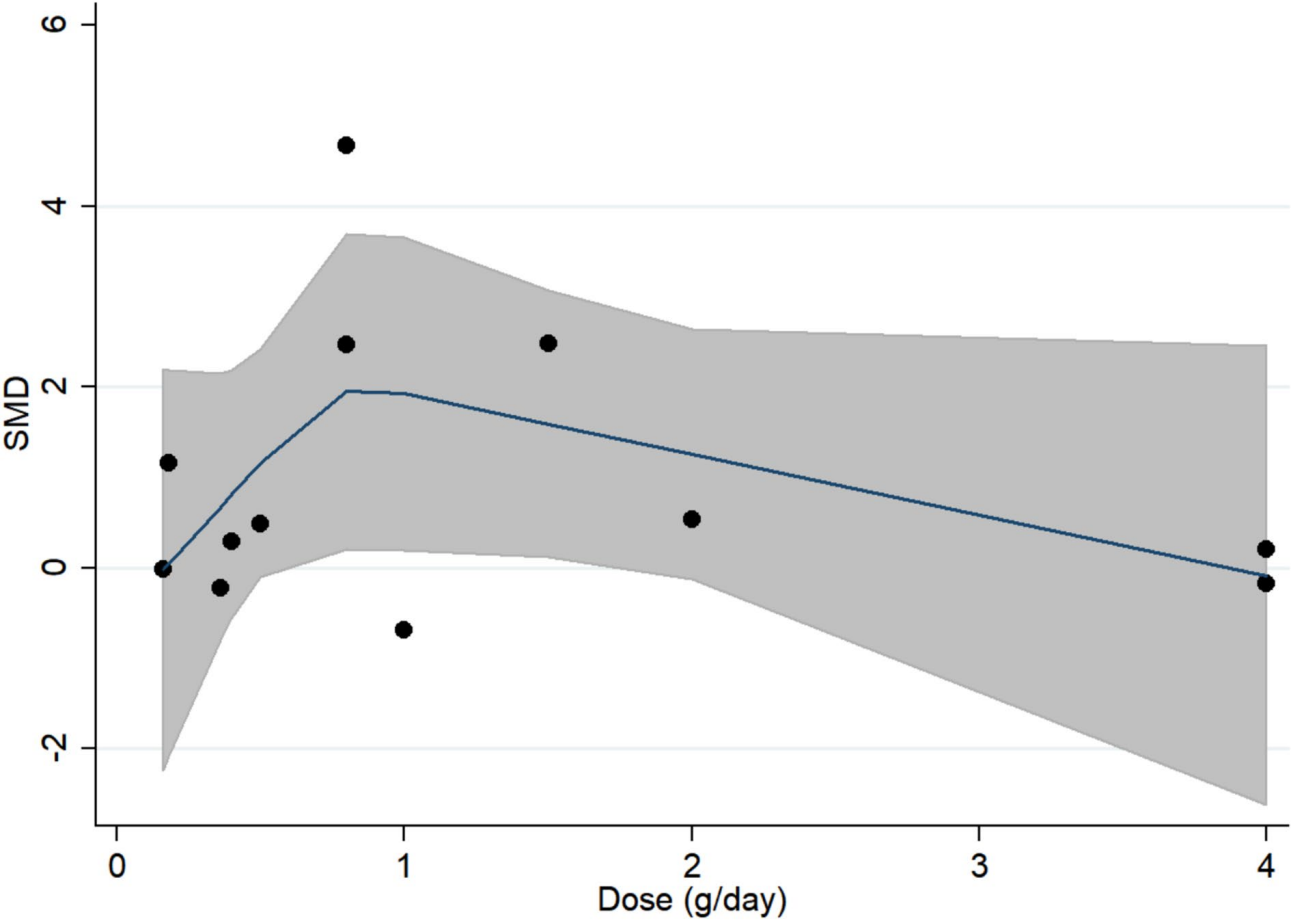
Restricted cubic spline analysis for daily dose of curcumin and global cognition.

The curvilinear dose–response relationship was shown in Figure 6. Curcumin achieved the best improving effect on global cognition at the dose of 0.8 g/day. When the dose was ≤0.8 g/day, the effect size was positively associated with daily dose (coefficient, 4.86; 95% CI, 0.09 to 9.63; *p* = 0.047). However, when the dose was ≥0.8 g/day, negative association was observed although non-significant (coefficient, −0.73; 95% CI, −1.98 to 0.52; *p* = 0.194) (Figure 6).

### Subgroup analysis

Significant improving effect of curcumin on cognitive function was observed in the following subgroups: duration ≥24 weeks (SMD, 1.15; 95% CI, 0.13–2.18; *p* = 0.027); age ≥ 60 years (SMD, 1.12; 95% CI, 0.03–2.21; *p* = 0.044); Asian countries (SMD, 0.96; 95% CI, 0.08–1.83; *p* = 0.032); curcumin product with enhanced bioavailability (SMD, 1.57; 95% CI, 0.29–2.84; *p* = 0.016) (Table 2). Otherwise, this effect became non-significant (*p* > 0.05).

**Table 2:** Subgroup analysis for the effect of curcumin on global cognition.

Subgroup analysis	*N*	SMD (95% CI)	*P*	*I*^2^ (%)
Duration				
≥24 weeks	8	1.15 (0.13, 2.18)	0.027	90.6
<24 weeks	4	0.22 (−0.21, 0.65)	0.321	52.6
Age				
≥60 years	8	1.12 (0.03, 2.21)	0.044	91.4
<60 years	4	0.31 (−0.04, 0.67)	0.085	37.5
Percentage of male				
≥50%	4	1.93 (−0.11, 3.97)	0.064	91.6
<50%	7	0.46 (−0.36, 1.28)	0.267	89.8
Not reported	1	0.29 (−0.16, 0.74)	0.202	NA
Healthy status				
AD	6	1.08 (−0.25, 2.41)	0.111	86.0
Schizophrenia	2	0.32 (−0.10, 1.64)	0.633	67.2
Healthy older adults	1	2.48 (1.94, 3.02)	<0.001	NA
Women with premenstrual syndrome and dysmenorrhea	1	0.49 (0.12, 0.86)	0.009	NA
Chemotherapy-induced cognitive impairment	1	0.29 (−0.16, 0.74)	0.202	NA
Overweight or obesity	1	−0.01 (−0.50, 0.47)	0.958	NA
Country				
Asian countries	6	0.96 (0.08, 1.83)	0.032	86.6
Western countries	6	0.69 (−0.36, 1.74)	0.199	91.4
Methods for evaluating cognitive function				
MMSE	4	1.51 (−0.70, 3.71)	0.180	91.1
MCCB	2	0.32 (−1.00, 1.64)	0.633	67.2
ADAS-Cog	2	0.36 (−0.40, 1.11)	0.354	0.0
MoCA	2	1.38 (−0.77, 3.53)	0.208	97.3
Others	2	0.26 (−0.22, 0.75)	0.289	61.5
Enhanced bioavailability or not of curcumin product				
Yes	5	1.57 (0.29, 2.84)	0.016	94.0
No	7	0.31 (−0.21, 0.84)	0.238	62.5

SMD, standard mean difference; MMSE, Mini-Mental State Exam; MCCB, MATRICS Consensus Cognitive Battery; ADAS-Cog, Alzheimer’s Disease Assessment Scale-Cognitive Subscale; MoCA, Montreal Cognitive Assessment; AD, Alzheimer’s Disease; NA, not available. Western countries in the present study include the United States and Australia; no included study was conducted in Europe.

### Sensitivity analysis

**Figure 7:**
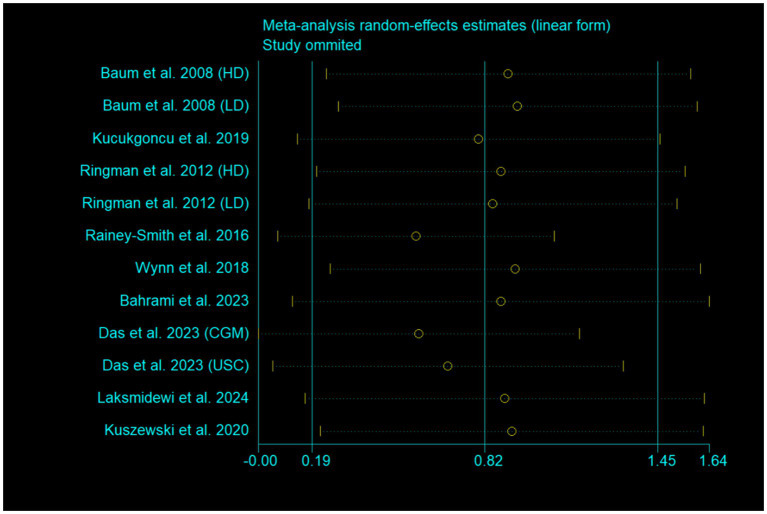
Sensitivity analysis by excluding each study one by one.

One study [Ringman et al. (16)] used both ADAS-Cog and MMSE to evaluate global cognitive function. Data from ADAS-Cog was used to generate pooled effect size in Figure 4. When data from MMSE was used, the pooled effect size was marginally significant (SMD, 0.65; 95% CI, −0.02 to 1.32; *p* = 0.056; *I*^2^ = 89.9%). The exclusion of each study one by one from the meta-analysis model did not obviously influence the pooled effect size, and all the results still remained significant (Figure 7). After excluding two studies with high risk of bias (15, 21), the pooled effect size was still significant (SMD, 0.63; 95% CI, 0.01 to 1.25; *p* = 0.046; *I*^2^ = 79.8%).

### Publication bias

**Figure 8:**
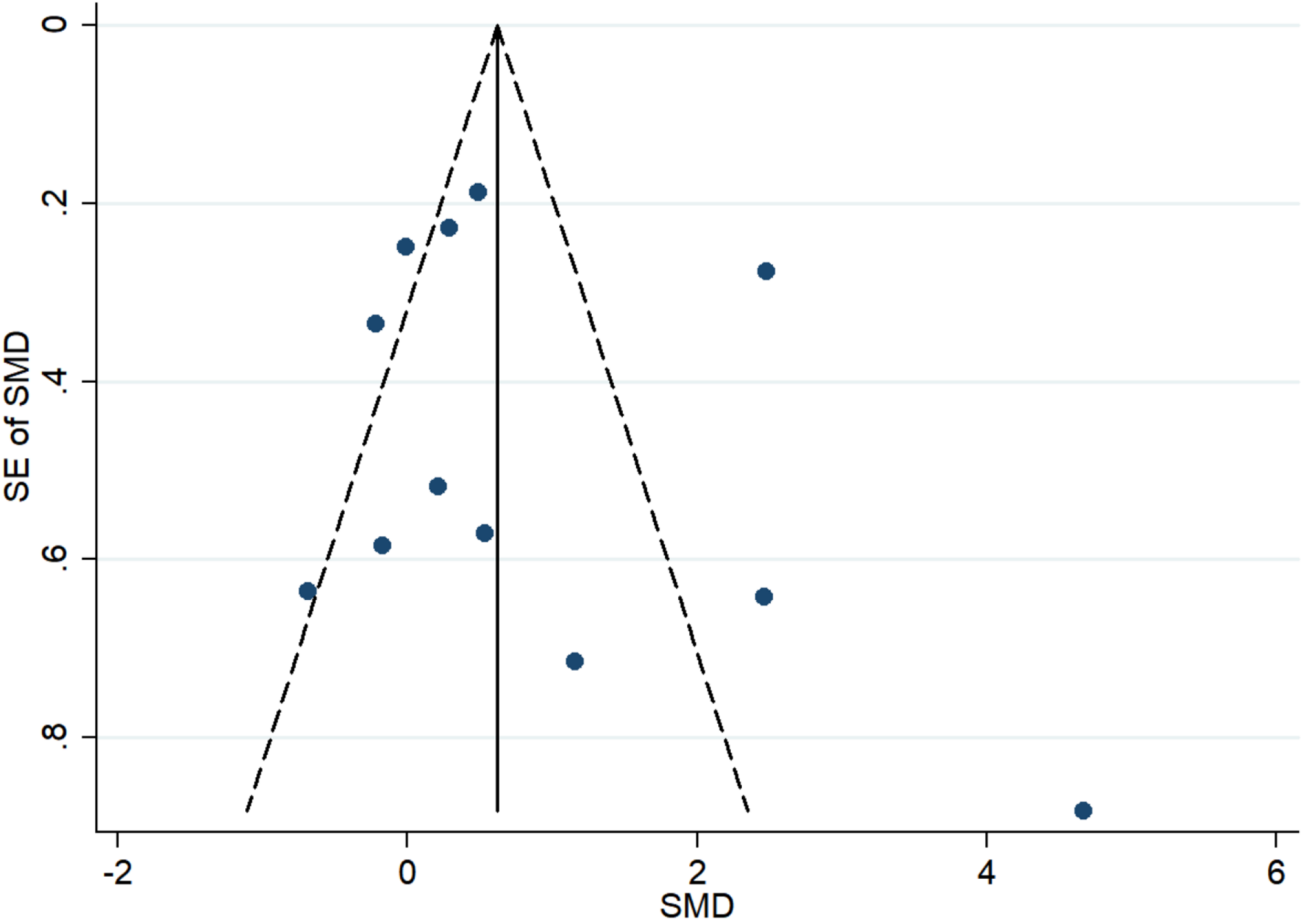
Funnel plot for publication bias.

The funnel plot was symmetric (*P* for Begg’s Test and Egger’s test = 0.150 and 0.493, respectively), indicating that there was no obvious publication bias (Figure 8).

## Discussion

In the present study with 501 subjects, supplementation of curcumin improved global cognition. Two previous meta-analyses of RCTs on this topic reported a non-significant overall effect size (18, 19). The number of subjects for evaluating global cognition in these two meta-analyses was only about half of the present study. Difference in sample size may be one possible reason for this inconsistency. The association of curcumin intake and improved cognition was also observed in cohort studies (8, 9). In addition, the present meta-analysis observed a non-linear dose–response relationship, and the optimal dose of curcumin was 0.8 g/day for improving global cognition. Curcumin has been reported to have a hormetic effect, and its some effects is greater at lower doses (27). Studies in mice indicated that curcumin had a better improving effect on AD at a lower dose (28). That may be why the improving effect of curcumin on global cognition tends to become weaker when dose is more than 0.8 g/day. Difference in dose is a source for between-study heterogeneity and can help explain the contradictory results observed in different RCTs. None of the two previous meta-analyses reported the dose–response relationship between curcumin and cognition. Low bioavailability of curcumin has been thought as a hinderance for its use as a therapeutic agent (29). Previous retrospective study indicated that Theracurmin (a curcumin formulation with very high absorbability) can successfully delay the progression of cognitive decline in subjects with mild cognitive impairment and AD (30). Subgroup analysis according to curcumin product having enhanced bioavailability or not observed consistent result. This indicates that curcumin formulation with high bioavailability may be considered as a therapeutic option for preventing or improving AD. In addition, difference in bioavailability of curcumin product is also an important source of between-study heterogeneity.

Subgroup analysis indicated that curcumin improved global cognition in subjects aged ≥60 years but not younger participants. The beneficial effect of curcumin on cognition in older subjects was also observed in previous meta-analysis in 2019 (19). However, contradictory result was found in AD subjects. Previous meta-analysis in 2019 reported a worse global cognition in AD subjects receiving curcumin (19), but the result became non-significant in the present study. The meta-analysis in 2019 included two RCTs in AD subjects, and the dose of curcumin ranged from 1 to 4 g/day (10, 16). Compared with the meta-analysis in 2019, the present meta-analysis included one more RCT of AD subjects, and its dose was 0.8 g/day (22). This RCT reported a significant improvement of global cognition after curcumin supplementation. Previous studies attributed the inconsistency to concomitant consumption of AD drugs, the severity of baseline disease and limited bioavailability of curcumin (10, 16). Despite this, difference in dose is another possible reason for the inconsistent results, considering the non-linear dose–response effect of curcumin on cognition discussed above. Due to limited bioavailability of curcumin (19) and weaker effect of too-high dose of curcumin, a longer duration of supplementation may be a good solution to obtain satisfactory effect. Subgroup analysis indeed observed that curcumin significantly improved global cognition only if duration was ≥24 weeks. In addition, subgroup analysis found that curcumin is more effective in Asian subjects than European subjects. We assume that genetic difference across ethnic groups is one possible reason (31, 32). Different ethnic groups also have different or even opposite response to some other nutrients, such as n-3 polyunsaturated fatty acids in patients with type 2 diabetes (33). Different health status of participants is another potential source of heterogeneity. Subgroup analysis observed improving effect in women with premenstrual syndrome and dysmenorrhea and healthy older subjects, and both subgroups included only 1 comparison and used highly bioavailable curcumin product (15, 21). Although the combined effect size for AD subjects was non-significant, but one included comparison observed beneficial effect of highly bioavailable curcumin on AD (22). Network pharmacology identified a series of targets for curcumin against AD (34). These evidence above indicated that curcumin with high bioavailability may improve cognition in AD, women with premenstrual syndrome and dysmenorrhea, and healthy older subjects. No beneficial effect of curcumin on cognition was observed in subjects with schizophrenia, chemotherapy-induced cognitive impairment, overweight or obesity, and more well-designed RCTs are needed to verify this result. Therefore, caution must be exercised when generalizing the results to these populations.

The improving effect of curcumin on global cognition has a biological basis. Curcumin is a polyphenol having strong antioxidant and anti-inflammatory activity (35, 36). AD pathology is linked to free radical damage, which acts as a signal contributing to amyloid-*β* and tau protein interactions (3). Previous evidence indeed indicated that curcumin can decrease the formation of amyloid-β plaques and increase its decomposition, decrease tau phosphorylation and increase its clearance rate (29). Experimental, epidemiological, neuropathological and genetic studies suggested a key role of immune activation and neuroinflammation in the pathology of AD (2). Latest evidence implies that curcumin may improve cognition by gut-brain axis and epigenetic modification (37, 38). Other mechanism may involve its suppression of acetylcholinesterase, acceleration of nerve repair, modulation of microglia activity, and regulation of miRNA activity (29, 39).

The present study had several strengths. Firstly, the sample size of the present study was about 2 times of previous meta-analyses, making the result more plausible. Secondly, dose–response analysis and subgroup analysis was conducted. The results provided us valuable information about factors that might influence the final effect of curcumin on cognition. The present study also had several limitations. Previous meta-analysis in 2021 evaluated the effect curcumin on both global cognition and different cognitive domains (such as working memory and proceeding speed) (18). Because only one additional RCT on different cognitive domains was published since 2021 (20), we did not conducted updated meta-analysis for results of cognitive domains. Secondly, a risk of bias was observed in 2 included studies. But sensitivity analysis showed that these biases did not have a significant influence on the pooled effect size. In addition, the dose range of included studies had a large span. However, only 12 comparisons were included in the dose–response analysis, which may influence the accuracy for estimating the optimal dose.

## Conclusion

Supplementation of curcumin can effectively improve global cognitive function, and the optimal dose is 0.8 g/day. The beneficial effect of curcumin on cognition is more obvious in older and Asian participants than younger and Western ones. The duration of supplementation is recommended to be more than 24 weeks for improving global cognition.

## Data Availability

The original contributions presented in the study are included in the article/Supplementary material, further inquiries can be directed to the corresponding authors.
